# eIF3 Interacts with Selenoprotein mRNAs

**DOI:** 10.3390/biom12091268

**Published:** 2022-09-09

**Authors:** Hassan Hayek, Gilbert Eriani, Christine Allmang

**Affiliations:** 1 Architecture et Réactivité de l’ARN, Université de Strasbourg, Centre National de la Recherche Scientifique, Institut de Biologie Moléculaire et Cellulaire, 67084 Strasbourg, France; 2 Department of Microbiology, Immunology, and Inflammation, Center for Inflammation and Lung Research, Temple University, Philadelphia, PA 19140, USA

**Keywords:** eukaryotic initiation factor, selenoprotein mRNA, RNA-protein interaction, translation regulation, GPx1

## Abstract

The synthesis of selenoproteins requires the co-translational recoding of an in-frame UGASec codon. Interactions between the Selenocysteine Insertion Sequence (SECIS) and the SECIS binding protein 2 (SBP2) in the 3′untranslated region (3′UTR) of selenoprotein mRNAs enable the recruitment of the selenocysteine insertion machinery. Several selenoprotein mRNAs undergo unusual cap hypermethylation and are not recognized by the translation initiation factor 4E (eIF4E) but nevertheless translated. The human eukaryotic translation initiation factor 3 (eIF3), composed of 13 subunits (a-m), can selectively recruit several cellular mRNAs and plays roles in specialized translation initiation. Here, we analyzed the ability of eIF3 to interact with selenoprotein mRNAs. By combining ribonucleoprotein immunoprecipitation (RNP IP) in vivo and in vitro with cross-linking experiments, we found interactions between eIF3 and a subgroup of selenoprotein mRNAs. We showed that eIF3 preferentially interacts with hypermethylated capped selenoprotein mRNAs rather than m^7^G-capped mRNAs. We identified direct contacts between GPx1 mRNA and eIF3 c, d, and e subunits and showed the existence of common interaction patterns for all hypermethylated capped selenoprotein mRNAs. Differential interactions of eIF3 with selenoprotein mRNAs may trigger specific translation pathways independent of eIF4E. eIF3 could represent a new player in the translation regulation and hierarchy of selenoprotein expression.

## 1. Introduction

Selenoproteins are mainly antioxidant enzymes that play important roles in the prevention of cancers, stress-related pathologies, and resistance to viral infections [1,2]. They are characterized by the presence of a selenocysteine in their active site. In humans, 25 selenoprotein genes have been identified [3,4]. Well-characterized members include glutathione peroxidases (GPx1, GPx4), thioredoxin reductases (TRXNRD1), iodothyronine deiodinases (DIO), methionine sulfoxide reductases (MSRB1), and endoplasmic reticulum selenoproteins (SELENOF, SELENOS, SELENOK, SELENON, and SELENOM) [5,6].

Translation of selenoprotein mRNAs requires the insertion of selenocysteine (Sec) by recoding an in-frame UGA codon usually decoded as a stop signal and involves a set of dedicated *cis-* and *trans*-acting factors [7,8]. In mammals, this process relies on the assembly of RNA–protein (RNP) complexes to specific stem-loops located in the 3′untranslated region (3′UTR) of selenoprotein mRNAs, called Selenocysteine Insertion Sequences (SECIS) [9,10,11]. The SECIS binding protein 2 (SECISBP2) interacts with the SECIS RNA and enables the recruitment of components of the selenocysteine insertion machinery to the mRNP [8,12,13]; amongst them, the specialized elongation factor eEFSec is in complex with the selenocysteinyl-tRNA^Sec^ [14,15]. The structure of the mammalian ribosome as it decodes the selenocysteine UGA codon was recently obtained by cryo-electron microscopy [16]. SBP2-SECIS forms a docking site on the ribosome for eEFSec/GTP/Sec-tRNASec that is stabilized by ribosomal protein eS31 [16]. Other SECIS-RNA-binding proteins, such as the ribosomal protein L30, translation initiation factor 4A3 (eIF4A3), and nucleolin, have been identified as modulators of selenoprotein synthesis [17,18,19]. Correct assembly of SBP2 and SECIS-binding proteins to the 3′UTR of selenoprotein mRNAs is a pre-requisite for their translation and was suggested to occur in the nucleus [15,20]. We have shown that the assembly of selenoprotein mRNPs is similar to that of several small non-coding RNPs such as spliceosomal snRNPs (small nuclear RNPs) and snoRNPs (small nucleolar RNPs), and that they share the same assembly machinery [21,22,23,24]. SBP2 associates with the Hsp90-cochaperone R2TP (Rvb1, Rvb2, Spagh, and Pih1)-Nufip complex. This machinery controls mRNP assembly of the L7Ae family that includes SBP2 [22]. We revealed another similarity between these mRNAs and showed that the cap hypermethylation enzyme trimethylguanosine synthase 1 (Tgs1) was recruited to selenoprotein mRNAs [23]. The assembly chaperone survival of motor neuron (SMN) plays a major role in this process by interacting with both SBP2 and Tgs1 [24].

Strikingly, several mammalian selenoprotein mRNAs, including MSRB1, GPx1, GPx4, SELENOM, and SELENOW, undergo 5′-end maturation, converting the canonical m^7^ guanosine cap into 2,2,7-trimethylated guanosine cap (m_3_^2,2,7^G or TMG cap) [23]. These mRNAs are the first examples of m_3_^2,2,7^G-capped mRNAs characterized in mammals [23,24]. Recognition of the 5′ cap of mRNAs by the initiation factor eIF4E that is part of the translation initiation complex eIF4F (composed of the three subunits eIF4E, eIF4A and eIF4G) constitutes classically the first step of canonical translation initiation and is highly specific for the m^7^G cap. Consistently, selenoprotein mRNAs with hypermethylated caps are not recognized efficiently by the translation initiation factor eIF4E but they are nevertheless actively translated because they were found associated to polysomes [23]. Moreover, the in-vivo translation of GPx1 is sensitive to the downregulation of Tgs1, but not to that of translation initiation factor eIF4E. The m_3_^2,2,7^G cap does not occur on all selenoprotein mRNAs, this modification could possibly contribute to hierarchy of selenoprotein expression by modulating eIF4E-dependent translation initiation. How hypermethylated capped selenoprotein mRNAs are translated remains to be uncovered. Interestingly, GPx1 and GPx4 mRNAs were identified in a genome-wide analysis among mRNAs that use non-canonical modes of initiation and whose translation is resistant to torin, an inhibitor that interferes with the interaction between eIF4E and eIF4G1 [25].

Mechanisms of cap-dependent translation that are independent of the eIF4E/m^7^G have been identified and suggested by multiple transcriptomic analyses [26,27,28,29]. Some of these selective modes of translation initiation that bypass canonical translation checkpoints involve the eukaryotic translation initiation factor 3 (eIF3) [30]. Interestingly, a cap-dependent mechanism, involving the eukaryotic translation initiation factor 3 (eIF3) as substitute of eIF4E for the cap recognition, has been found [31], and eIF3 was shown to target specific mRNAs for translation regulation.

The eukaryotic translation initiation factor 3 (eIF3) is a large 800 kDa complex of 13 subunits (eIF3a-eIF3m) that play crucial roles in organizing initiation factors and ribosome interactions during translation initiation, termination, and recycling [32,33,34]. Multiple steps of the translation initiation process are dependent on eIF3, including stabilization of eIF2/GTP/Met-tRNA_i_^Met^ ternary complex binding to 40S subunits, recruitment of 5′ capped mRNAs to 40S subunits, and assistance in scanning of the 5′UTR for AUG codon recognition. eIF3 also acts as a translational activator or repressor by binding to RNA structures in the 5′UTRs of specific mRNAs [29,35,36,37]. The spatial organization of mammalian eIF3 is based on a conserved multilobed octamer that is also found in proteasome and signalosome complexes. Six eIF3 subunits (a, c, e, k, l, and m) have PCI domains (Proteasome, COP9, eIF3), and two subunits (f, h) have MPN domains (Mpr1–Pad1 N-terminal). Different cryo-EM models have been proposed [38,39]. eIF3d is located in a more peripheral position; it interacts both with the 40S and the octameric core, as well as potentially with eIF4F [40]. eIF3d is not required for the integrity of the complex and not conserved across species but is essential in some organisms. eIF3d was also shown to bind the 5′ cap of some specific mRNAs in a way reminiscent of eIF4E, suggesting the existence of a second mechanism of cap-dependent translation linked to eIF3d [28,31]. Other peripheral proteins include the eIF3 b, g, and i module that encircles the 40S and is believed to connect the mRNA entry channel to the exit site of the ribosome [38,39,41]. Most subunits are engaged in multiple functional interactions. eIF3c for instance is a crucial binding partner of eIF1 and eIF5 and its NTD extends into the 40S decoding centre proximal to eIF1 [42]. The specific contribution of individual subunits to the variety of eIF3 functions has only started to be elucidated. Interestingly, eIF3 was also shown to participate in programmed stop readthrough; this role being conserved between yeast and humans [43]. Its role in selenoprotein synthesis and UGA_sec_ recoding has never been explored.

Here we report that eIF3 binds directly to a subset of selenoprotein mRNA. Combining ribonucleoprotein immunoprecipitation (RNP IP) in vivo and in vitro and cross-linking we found that eIF3 interacts preferentially with the hypermethylated capped selenoprotein mRNAs. We demonstrate direct interactions of GPx1 mRNA with eIF3 c, d, and e subunits in vitro and show the existence of common interaction patterns for all the hypermethylated capped selenoprotein mRNAs that interact with eIF3. Selenoprotein mRNAs were demonstrated to be differentially processed, regulated, and translated. Here we show that they also interact differentially with initiation factors since not all selenoprotein mRNAs are directly bound by eIF3. eIF3 could therefore represent a new player in the translation regulation of selenoprotein mRNAs.

## 2. Materials and Methods

### 2.1. Cell Culture

HEK293FT cells were cultured at 37 °C in 5% CO_2_ in Dulbecco’s Modified Eagle Media (DMEM) containing 10% fetal calf serum (FCS), 1% penicillin-streptomycin (Invitrogen, Illkirch, France), and 500 µg/mL geneticine. RNP complexes were stabilized by formaldehyde cross-linking. Cells were washed with DPBS (Gibco, Illkirch, France) and the pellets were resuspended in 1 vol of 0.2% formaldehyde for 5 min. Cross-linking reactions were quenched by the addition of 0.15 M glycine pH 7 for 5 min. Cell extracts were prepared in RNP buffer (10 mM HEPES-NaOH pH 7.9, 100 mM KCl, 5 mM MgCl_2_, 0.5% NP-40, 1 mM DDT, 100 U/mL RNasin (Promega, France), 400 µM Vanadyl Ribonucleotide Complex (VRC) (Sigma, Darmstadt, Germany), and anti-protease cocktail (Sigma, Darmstadt, Germany).

### 2.2. Immunopurification, RT-PCR and Western Blotting

Endogenous eIF3 complexes were immunopurified from HEK293FT cells as described in [35]. Three hundred microliters of cell extracts was diluted in Lysis Buffer (50 mM Tris-HCl pH 8, 150 mM NaCl, 1% Triton X-100) and incubated with 100 μL of protein A μMACS magnetic beads (Miltenyi, Bergisch Gladbach, Germany) charged with 2 µg of anti-eIF3b antibody (Bethyl, Montgomery, TX, USA, A301-761A). Beads were washed four times and eluted in Laemmli buffer according to the manufacturer’s instructions. Proteins were analyzed by Western blotting using the antibodies listed in Table S1. Bound RNA was extracted by phenol/chloroform and precipitated. After DNase treatment, RNAs were reversed transcribed using AMV-RT (Q-Biogen, Illkirch, France). Levels of mRNAs were measured by RT-PCR (qRT-PCR). Reactions were carried out on a CFx94 (Bio-Rad, Marnes-la-Coquette, France) using the Maxima SYBR Green PCR kit (Fermentas, Illkirch, France). Oligonucleotides used for qRT-PCR are listed in Table S2. qRT-PCR reactions were performed and designed according the MIQE guidelines [44], the specificity of the oligonucleotides was validated, and the amplification efficiencies of the primer sets are all between 90 and 110% and r^2^ values above 0.98. The % of RNAs in the IP were calculated according to the ∆Cq method and normalized by the input RNAs. Results were expressed as mean ± standard error of an average of three measurements.

### 2.3. ThioU GPx1 mRNA-eIF3 Cross-Linking Reactions and 2D Gel Analysis

4-thioU and [α-^32^P]-ATP labeled GPx1 mRNA were synthesized by T7 RNA polymerase in-vitro transcription of a DNA template generated by PCR as described previously [35,45]. For cross-linking reactions, radiolabeled ThioU-GPx1 mRNA (50,000 cpm) were incubated in the presence of 5 μM of purified eIF3 protein complex in a final volume of 4 μL of cross-linking buffer (100 mM KCl, 20 mM Tris HCl, pH 7.5, 1 mM DTT, 0.1 mM EDTA, 10% glycerol) for 30 min at 25 °C. The ThioU mRNA-protein cross-linking reaction was performed by UV 365 nm irradiation for 30 min. RNase A (Roche, Mannheim, Germany) digestion was done for 30 min at 37 °C to degrade mRNA fragments not protected by eIF3 subunits. Radioactive mRNA fragments cross-linked to eIF3 subunits remain bound to the proteins.

To identify eIF3 subunits interacting with GPx1 ThioU mRNA, cross-linking reactions were analyzed by 2D gel electrophoresis followed by Western blot as described in details in [35]. Samples were separated by isoelectric focusing (IEF) in the first dimension (pH 4–7) using ReadyStripTM IPG Strips (BioRad, Marnes-la-Coquette, France) in a Protean IEF Cell generator (BioRad) according to the manufacturer’s conditions. Separation in the second dimension was performed on a 10% SDS-PAGE in TGS buffer (25 mM Tris-HCl, pH 8.8, 200 mM glycine, 0.1% SDS). Radiolabeled proteins were transferred to an Immobilon-P membrane (Millipore, Molsheim, France). The membrane was scanned by Phosphorimaging and subjected to Western blot analysis using the ChemiDoc imaging system (BioRad); 2-D radioactivity and Western blot images were overlaid.

### 2.4. Recombinant Proteins and GST Pull-Down Assays

Recombinant HisGST-tagged eIF3c truncated proteins: HisGSTeIF3d, HisGSTeIF3e, and HisGSTeIF3g were expressed in *E. coli* and purified using Ni-NTA agarose (Qiagen, Hilden, Germany) by standard procedure. For GST pull-down experiments, 40 µg of purified HisGST proteins were bound to 50 µL of GST-Trap agarose beads (Chromotek, Planegg, Gremany) and incubated with 60 µg of HEK293FT total RNA in binding buffer (20 mM Tris-HCl pH 7.5, 100 mM KCl, 0.1 mM EDTA, 1 mM DTT, 10% glycerol, 400 μM VRC, 100 U RNasin/mL, anti-protease cocktail) for 30 min at 4 °C. Beads were washed 3 times with binding buffer. Total RNAs present in the flow through or on the beads were extracted by phenol/chloroform and ethanol precipitated. Specific RNAs were then quantified by qRT-PCR as previously described.

## 3. Results

### 3.1. eIF3 Interacts In Vivo with a Subclass of Selenoprotein mRNAs

**Figure 1:**
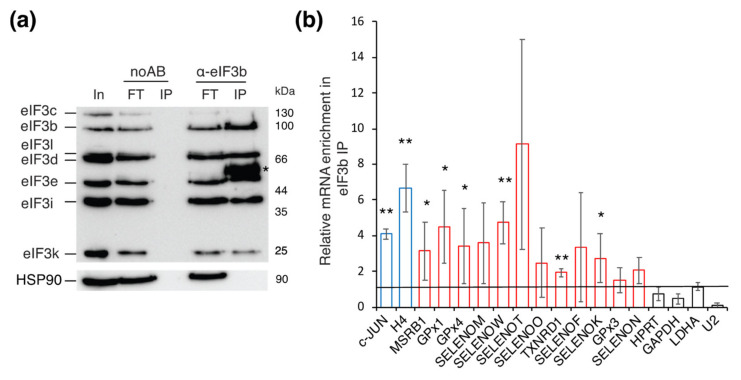
eIF3 interacts in vivo with selenoprotein mRNAs. Immunoprecipitation of the endogenous eIF3 complex and its associated RNAs from HEK293FT cells using antibodies against eIF3b (α-eIF3b). (**a**) Analysis of the endogenous immunoprecipitated proteins by SDS-PAGE and Western blotting using antibodies against the indicated proteins. In: Input (5% of total); (FT): Flow through; (IP) immunoprecipitation beads; noAB: control without antibodies. The position of the molecular weight markers is indicated. * Corresponds to the anti-eIF3b antibodies released from the immunoprecipitation beads and detected by the secondary antibodies by Western blotting (**b**) qRT-PCR analysis of eIF3-interacting mRNAs. The relative mRNA enrichment in the eIF3b IP was measured by qRT-PCR and determined by the *∆∆Ct* method using LDHA mRNA as a normalizer. c-JUN and histone H4 mRNAs (blue bars) are targets of eIF3 according to Lee et al., (2015) and Hayek et al., (2021) respectively. MSRB, GPX1-4, TRXNRD1, and SELENO are mRNAs of selenoproteins (red bars). HPRT (hypoxanthine guanine phosphoribosyltransferase), GAPDH (glyceraldehyde-3-phopshate dehydrogenase), and LDHA (lactate dehydrogenase A) are canonical mRNAs, and snRNA U2 is a negative control (black bars). Error bars represent the standard deviation of three independent sets of immunoprecipitation experiments, each followed by triplicate qRT-PCR measurements. The horizontal line represents the level of LDHA mRNA binding (5% in average, normalized to 1). Asterisks indicate statistically significant differences with the LDHA control mRNA. * *p* < 0.05 and ** *p* < 0.005 based on Student’s *t* test.

To determine if eIF3 is capable to interact with selenoprotein mRNAs, we immunoprecipitated eIF3-RNA complexes from HEK293FT cells. HEK293FT cells express a wide range of selenoproteins and have been validated for both selenoprotein mRNA regulation studies and eIF3-RNA complex isolation [11,23,24,35]. The full endogenous eIF3 complex was immunoprecipitated using antibodies directed against the eIF3b subunit [29,35]. We performed anti-eIF3b immunoprecipitation in conditions of formaldehyde cross-linking to stabilize interactions within the eIF3 complex and minimize RNP complexes rearrangements as described previously [35]. The eIF3 subunits exist at equal stoichiometry within the complex and are tightly associated in human, except for eIF3j, which is loosely bound and non-essential for viability in several species. The eIF3 nucleation core formed by the eIF3a and eIF3b subunits is a prerequisite to link the rest of the PCI core subunits and the peripheral eIF3b, g, and i module [34]. Western Blotting confirmed that representative octameric core eIF3 proteins (c, e, k, and l), peripheral eIF3 proteins (b and i), as well as the cap-binding protein eIF3d were specifically co-immunoprecipitated by anti-eIF3b (Figure 1a). It can therefore be assumed that all the eIF3 subunits were recovered in the immunoprecipitation. In addition, the relative intensity of the Western blot signals for the different subunits in the immunoprecipation remains comparable to that of the input, indicating that the apparent stoichiometry of the eIF3 complex is preserved (Figure 1a). No interaction was detected for HSP90 used as a negative control. In the absence of eIF3b antibodies, no complex was recovered and the eIF3 complex remained in the flow through (FT). RNAs bound to eIF3 were extracted and analyzed by quantitative RT-PCR (Figure 1b). For the detection of selenoprotein mRNAs, we used primers complementary to 12 out of the 25 selenoprotein mRNAs characterized in mammals. The c-JUN mRNA [29] and H4 mRNA [35] are direct targets of eIF3, they were used as positive controls. The housekeeping mRNAs HPRT (hypoxanthine guanine phosphoribosyltransferase), GAPDH (glyceraldehyde-3-phosphate dehydrogenase) and LDHA (lactate dehydrogenase A) undergo canonical cap-dependent translation [29,35]. The spliceosomal U2 snRNA was used as a negative control. The relative mRNA enrichment in the eIF3b IP was determined by qRT-PCR using LDHA mRNA as a normalizer. Similar results were obtained when data were normalized against the three housekeeping control mRNAs. Consistent with our previously published results, the c-JUN and H4 mRNA were co-immunoprecipitated by eIF3 and enriched 4–6,6 times in the IP, whereas the control mRNAs HPRT and GAPDH were not [29,35]. Our results indicate that selenoprotein mRNAs are enriched 4-fold on average in the anti-eIF3b immunoprecipitation (Figure 1b). This is comparable to the results obtained for c-JUN and H4 mRNAs. Two groups of selenoprotein mRNAs seem to emerge. Seven selenoprotein mRNAs, namely MSRB1, GPx1, GPx4, SELENOM, SELENOW, TXNRD1, and SELENOK are significantly enriched in the anti-eIF3b immunoprecipitation compared to LDHA (Figure 1b). The binding of the five other selenoprotein mRNAs was not significant (SELENOT, SELENOO, SELENOF, GPx3, and SELENON), although eIF3 binding was globally increased (Figure 1b). Taken together, these results suggest that selenoprotein mRNAs are targets of eIF3 but exhibit differential binding patterns to eIF3. Strikingly, the group of selenoprotein mRNAs that bind eIF3 also belongs to the class of selenoprotein mRNAs that have a hypermethylated cap and are not efficiently recognized by the translation factor eIF4E [23].

### 3.2. Identification of eIF3 Subunits That Interact with GPx1 mRNA by In Vitro Cross-Linking

**Figure 2:**
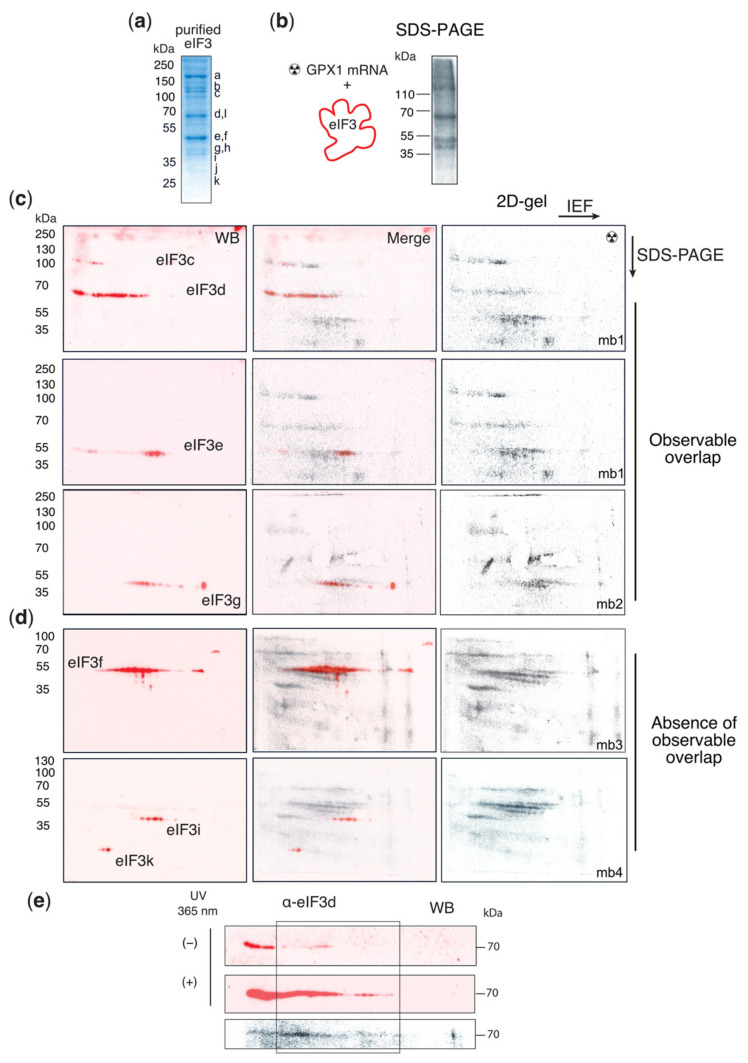
Identification of eIF3 subunits c, d, e, and g in interaction with GPX1 mRNA by UV crosslinking and Western blotting. (**a**) SDS-PAGE gel showing the purified eIF3 complex obtained from W.C Merrick [47]. The subunits are labeled according to their approximate mobilities. (**b**) UV crosslinking of [α^32^P]-ATP radiolabeled thioU-GPx1 mRNA in the presence of purified eIF3 complex at 365 nm. After RNase A digestion, radioactive mRNA fragments protected against degradation remain cross-linked to eIF3 subunits. [^32^P]-labeled proteins are resolved by SDS-PAGE (**b**) or 2D-gel electrophoresis (**c**). In the first dimension, proteins were separated by isoelectric focusing (IEF) pH 4–7 followed by SDS-PAGE in the second dimension. Radiolabeled proteins are transferred to PVDF membranes, revealed by Phosphorimaging (**b**). (**c**,**d**) Western blotting (WB) using antibodies directed against individual eIF3 subunits. Identification of cross-linked proteins by superimposition of WB signals (red signals, left panels) and radioactive signals (grey signal, left panels) are visible in the overlay (merge, middle panels). Three replicate experiments were performed. The PVDF membranes in panels (**c**) and (**d**) are products of four independent 2D gels. The PVDF membranes are numbered mb1–mb4. In panel (**c**), mb1 was probed successively with α-eIF3c, α-eIF3d and α-eIF3e, mb2 by α-eIF3g antibodies, the membrane mb3 of panel (**d**) with α-eIF3f, mb4 with α-eIF3i and α-eIF3k. The first ^32^P panels (mb1) are therefore duplicate images. The position of the molecular weight markers is indicated. (**d**) No eIF3j signal was detected and therefore no superimposition was reported between eIF3j and GPx1. (**e**) Comparison of the migration profiles of eIF3d on 2D gel in the presence (+) and absence (−) of GPx1 mRNA crosslinking. The cross-link of eIF3d to GPx1 mRNA induces a shift of the WB signal to the boxed area.

The eIF3 complex was shown to interact with its RNA targets with distinct combinations of eIF3 subunits [29]. We chose to decipher these interactions for the GPx1 selenoprotein mRNA. To identify individual eIF3 subunits within the large eIF3 complex that contacts GPx1 mRNA, we used a ThioU-RNA crosslinking approach coupled to two-dimensional gel analysis (2D-gel). This technique was successfully used in the case of H4 mRNA [35]. The uniformly [α-^32^P]-ATP radiolabeled and ThioU-GPX1 mRNA transcript (899 nts) was obtained by in-vitro transcription; it contained the full open-reading frame as well as complete natural 5′ and 3′ UTR sequences. In-vitro transcribed mRNAs containing 4-thiouridines allow to precisely decipher RNA protein interactions using purified components. This method allowed the characterization of interactions between mRNAs and ribosomal proteins as well as numerous translation initiation factors including eIF3 [17,31,35,45]. After refolding to promote the formation of secondary and tertiary structures, cross-linking of ThioU-GPX1 mRNA was performed in the presence of purified eIF3 complex (Figure 2). After digestion with RNase A, the eIF3 subunits cross-linked to the ^32^P-labelled mRNA fragment with which they interact were resolved by denaturing gel electrophoresis. Four of the thirteen eIF3 subunits with apparent molecular weights of 110, 65, 50, and 45 kD cross-linked directly to GPx1 mRNA (Figure 2a). Because several eIF3 subunits have similar molecular weight, separation of the cross-linked products by two-dimensional gel electrophoresis (2D-gel) (Figure 2b) followed by Western blot analysis was used for their identification, with the superposition of Western blot and radioactivity signals indicating crosslinking (Figure 2c). The results unambiguously reveal the existence of cross-linking between eIF3c, d, e, and g and GPx1 mRNA (Figure 2c). eIF3 subunits undergo numerous post-translational modifications [46], resulting in a unique dotted migration profile on the 2D-gel, reflecting the levels of modifications, and thus isoelectric charge of the protein (Figure 2c). This furthermore allows confident attribution of the protein crosslinks. For example, eIF3e, f, and g have similar molecular weights, but no crosslinking signal could be observed between eIF3f and GPx1 mRNA because Western blot and radioactivity signals do not overlay (Figure 2d), showing that eIF3f does not interact with GPx1 mRNA. The Western blot signals for the eIF3 subunits i and k do also not overlay with the radioactivity signal, showing that they do not interact with GPx1 mRNA. In the absence of cross-linking, the migration profile of the free subunits is different as shown in the case of the eIF3d (Figure 2e) and previously published [35]. After cross-linking, the migration profile of the proteins, here eIF3d (Figure 2e), is shifted left-to-right and spreads over a wide range of pH because of the presence of the additional negative charges coming from the cross-linked RNA moiety.

### 3.3. Differential Binding of eIF3c, d, e, and g Subunits to Selenoprotein mRNAs In Vitro

**Figure 3:**
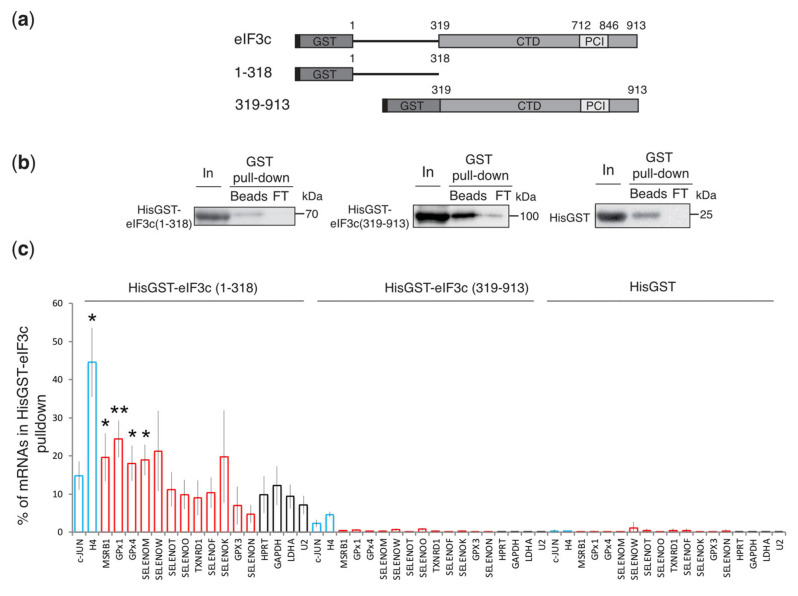
The N-terminal domain of eIF3c interacts with selenoprotein mRNAs. (**a**) Schematic representation of eIF3c (1-913) and truncated recombinant eIF3c fused to hexa-histidine and glutathione S-transferase (HisGST). eIF3c contains a PCI (Proteasome, COP9/signalosome, eIF3) motif (712-846) in the C-terminal region (CTD). eIF3c (1-318) corresponds to the N-terminal domain and eIF3c (319-913) to the CTD. (**b**) GST pull-down experiments were performed using the HisGST proteins and total RNA extracted from HEK293FT cells. HisGST alone was used as a control protein. These experiments were performed to analyze in parallel the binding of HisGST eIF3 subunits to histone mRNAs and selenoprotein mRNAs. The Western blots presented are thus the same as those previously published in [35]. Adapted with permission from Ref. [35]. Copyright 2021, CC BY License, copyright to The authors and American Society for Biochemistry and Molecular Biology. Western blot panels confirm binding of the HisGST target proteins to GST-trap matrix (Beads). In: Input (5% of total); Beads: 10% of bound protein; FT: 5% effluent. The position of the molecular weight markers is indicated to the right of the panels. (**c**) Bound RNAs were analyzed by qRT-PCR and are as described in Figure 1b. The histograms represent the % of mRNAs in GST pull-down compared to the input. Blue bars: c-JUN and H4 mRNAs; red bars: selenoprotein mRNAs; black bars: control HPRT, GAPDH, LDHA mRNAs, and U2 snRNA. The error bars represent the standard deviation of three biological replicates. Asterisks indicate statistically significant differences with the LDHA control mRNA. * *p* < 0.05 and ** *p* < 0.005 based on Student’s *t* test.

The ability of the cross-linked subunits to interact with selenoprotein mRNAs independently of the eIF3 complex was tested by glutathione-S-transferase (GST) pull-down experiments using HisGST eIF3c, d, e, and g proteins and total RNAs from HEK293FT cells. Soluble HisGSTeIF3c could not be expressed, so we produced the truncated HisGSTeIF3c (1-318) N-terminal and HisGSTeIF3c (319-913) C-terminal proteins (Figure 3a,b), as reported previously [48]. The HisGSTeIF3c (1-318) region has been shown to bind to histone mRNAs [35] as well as eIF5 and eIF1 proteins [35,48,49], while HisGSTeIF3c (319-913) contains a PCI (Proteasome, COP9/signalosome, eIF3) motif that mediates and stabilizes protein-protein interactions within the eIF3 complex [50,51]. The binding of 25 selenoprotein mRNAs to the HisGST eIF3 proteins was analyzed by qRT-PCR as described above (Figure 3c). C-JUN and H4 mRNAs were used as positive controls, while the housekeeping mRNAs HPRT, GAPDH, and LDHA were used as negative controls. None of the tested mRNAs interacted with the C-terminal eIF3c region (319-913), which is consistent with previous observations [35] and no mRNA interaction could be detected with a HisGST control protein (Figure 3c). In contrast, HisGST-eIF3c (1-318) preferentially interacted with MSRB1, GPx1, GPx4, SELENOM, SELENOW, and SELENOK, for which 18–24% of the mRNA was recovered in the bound fraction (Figure 3c). This value is within the range of the values observed for the positive controls C-JUN (15%) and H4 (45%) mRNAs. Six selenoprotein mRNAs (SELENOT, TXNRD1, SELENOF, SELENON, GPx3, and SELENOO) were pulled down to lower levels, comparable to the housekeeping mRNAs (10%) (Figure 3c).

**Figure 4:**
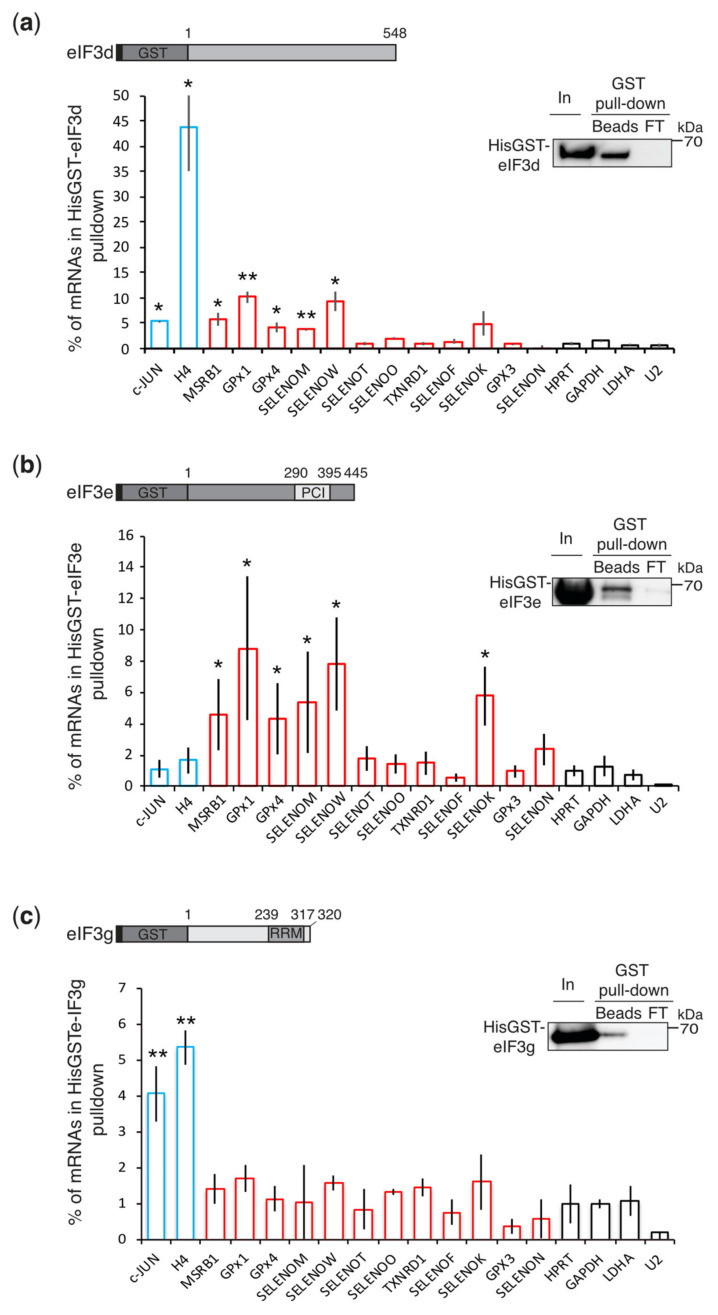
Interaction analysis of HisGSTeIF3 d, e, and g with selenoprotein mRNAs. (**a**–**c**) GST pull-down results for HisGST-eIF3d, HisGST-eIF3e, and HisGST-eIF3g and qRT-PCR analysis of bound RNAs. The schematic representation of the proteins and the Western blot panels confirming the pull-down of the HisGST target proteins are included above the histograms. eIF3e contains a C-terminal PCI domain and eIF3g a C-terminal RNA Recognition Motif (RRM). Data are presented as described in Figure 3. The GST pull-down experiments and the Western blots presented are the same as those previously published in [35]. Adapted with permission from Ref. [35]. Copyright 2021, CC BY License, copyright to The authors and American Society for Biochemistry and Molecular Biology. * *p* < 0.05 and ** *p* < 0.005 based on Student’s *t* test.

The same interaction pattern was observed for HisGST-eIF3d and HisGST-eIF3e (Figure 4a,b). Between 4 and 10% of MSRB1, GPx1, GPx4, SELENOM, SELENOW, and SELENOK mRNAs are bound by HisGST-eIF3d and HisGST-eIF3e. This is comparable to the binding levels of C-JUN mRNA to HisGST-eIF3d, while levels of H4 mRNA binding were 10 times higher as previously reported [35]. The interaction with HisGST-eIF3e appears to be specific to these selenoprotein mRNAs since only 2% of C-JUN, H4, and non-selenoprotein mRNAs are retained. The second group of selenoprotein mRNAs (SELENOT, TXNRD1, SELENOF, SELENON, GPx3, and SELENOO) were not bound by HisGST-eIF3d or HisGST-eIF3e (Figure 4a,b). This is consistent with the immunoprecipitation results of the endogenous eIF3 complex in vivo (Figure 1). In contrast, HisGSTeIF3g did not significantly interact with selenoprotein mRNAs since only 1% of mRNAs are retained, as it is the case for housekeeping control mRNAs (Figure 4c). Whereas 4–5% of C-JUN and H4 mRNAs interact with HisGSTeIF3g.

Overall, these results show that all the selenoprotein mRNAs that bind to eIF3 in vivo are recognized via direct interactions involving the eIF3 c, d, and e subunits in vitro. His-GSTeIF3g does not interact directly with selenoprotein mRNAs contrary to what was observed in our GPx1 mRNA cross-linking experiments. It is likely that the interaction between eIF3g and GPx1 mRNAs can only take place in the context of the entire complex. A common interaction pattern between the eIF3 complex and selenoprotein mRNAs is clearly emerging. This pattern is not the same for histone mRNAs and C-JUN mRNA, confirming earlier results showing that eIF3 mRNA targets interact with distinct combinations of eIF3a, b, d, e, and g subunits [29,35]. Not all selenoprotein mRNAs are recognized by eIF3, these results are in line with previous results showing that selenoprotein mRNAs also show differential binding to eIF4E, and are differentially processed, regulated, and translated [46,52,53].

## 4. Discussion

The rate-limiting event in selenoprotein expression is the translational recoding of UGA_Sec_. Selenium bioavailability and oxidative stress are global modulators of selenocysteine insertion efficiency. Transcript-specific regulators have also been reported to control selenoprotein hierarchy [53,54]. The type of SECIS and the sensitivity of the mRNA to NMD are important determinants for selenoprotein expression. SECIS binding proteins such as eIF4A3 and nucleolin play roles in this process. eIF4A3 is a negative regulator of GPx1, MSRB1, SELENON, and DIO1 [18], while nucleolin is a positive regulator of GPx4 and TRXNRD1 [55]. The choice between housekeeping (e.g., TRXNRD1, SELENOF, and GPx4) and stress-related selenoprotein mRNA expression (e.g., GPx1, GPx3, MSRB1 and SELENOT) can be made by methylation of the ribose of U34 of Ser-tRNA^[Ser]^^Sec^ and thus modulation of the ratio between two Sec-tRNA^[Ser]^^Sec^ isoforms [56]. Additionally, a subset of selenoprotein mRNAs (MSRB1, GPx1, GPx4, SELENOM, and SELENOW) undergo 5′-cap hypermethylation and are poorly recognized by eIF4E but nevertheless translated [46]. This suggests mechanisms of selective regulation at the level of initiation.

**Figure 5:**
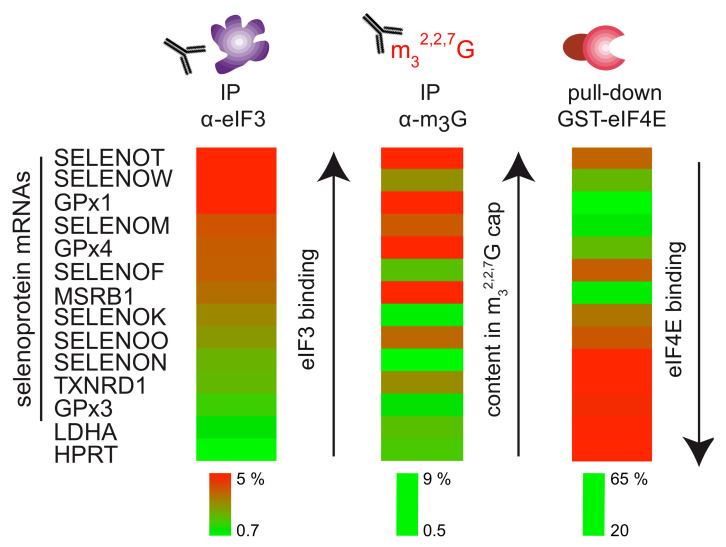
eIF3 binds mRNAs with hypermethylated caps that are poorly recognized by eIF4E. Heatmap representation of mRNA binding in anti-eIF3b IP is deduced from Figure 1b and compared to anti-m_3_^2,2,7^G IP and GST-eIF4E pull-down experiments from [23]. Adapted with permission from [23]. Copyright 2014, by-nc/3.0 licence, copyright to The authors and Oxford University Press. The binding scales are represented below the corresponding heatmaps. They represent the percentage of mRNAs in the bound fractions. Maximum binding values in each set of experiments are represented in red and minimal binding values in green. Heat maps were generated with the MeV software.

Here we showed that the multisubunit eIF3 factor can interact with a subset of selenoprotein mRNAs in vivo. Intriguingly, eIF3 preferentially binds to the group of selenoprotein mRNAs that are poorly recognized by eIF4E and have a hypermethylated cap (namely MSRB1, GPx1, GPx4, SELENOM, SELENOW, TXNRD1, and SELENOK). Combined with our previous data, results indicate an inverse correlation between eIF3 binding and eIF4E recognition, but a strong correlation with the presence of a m_3_^2,2,7^G cap on the target mRNA (Figure 5).

eIF3 has been involved in the selective translation of specific mRNA classes. In particular, mRNAs involved in cell proliferation as well as replication-dependent histone mRNAs [29,35]. Our findings suggest that eIF3 could also contribute to the selective regulation and hierarchy of selenoprotein expression.

We demonstrated that binding of eIF3 to GPx1 mRNA is mediated by the subunits eIF3c, d, e, and g that cross-link to GPx1 mRNA. We confirmed the existence of direct interactions between the subunits c, d, e, and GPx1 mRNA in vitro. eIF3 is composed of two interconnected modules. eIF3 subunits in contact with GPx1 mRNA are in both modules. The subunits eIF3c and e are part of the octameric core PCI subunits and are positioned near the mRNA exit channel [40]. eIF3g contains an RRM RNA-binding domain and was shown to be in contact with the mRNA entry channel [40]. Contacts between eIF3g and GPx1 mRNAs may be transient or only take place in the context of the entire eIF3 complex. eIF3d is a peripheral subunit attached to the octamer through eIF3e [33,57] but also eIF3c and eIF3a [40]. eIF3d is capable of binding to the cap of various mRNA [31]. Whether eIF3d could bind m_3_^2,2,7^G caps has not been studied. However, human regulatory T cells mRNAs were shown to recruit the eIF4G homolog DAP5 and eIF3d to their 5′UTR for non-canonical but cap-dependent translation mechanism [58]. Previous studies have revealed that distinct modes of interaction can exist between eIF3 and the RNAs it controls, these involve various combinations of eIF3a, b, d, g, and e subunits [29,35,48]. Our results suggest the existence of a common interaction pattern between the eIF3 complex and all the targeted selenoprotein mRNAs. Indeed, eIF3c, d, and e interact with selenoprotein MSRB1, GPx1, GPx4, SELENOM, and SELENOW mRNAs independently of the eIF3 complex. Among them, eIF3e was shown to form with eIF3d a module that orchestrates the expression of specific mRNAs involved in the control of cellular metabolism [59]. The eIF3d cap-binding protein is required for specialized translation initiation [31]. It was recently shown that eIF3d is activated by phosphorylation in response to metabolic stress in human cells, in particular during chronic glucose deprivation [60]. Thus, in conditions of canonical eIF4E translation inhibition, eIF3d-mediated cap-dependent translation allows the translation of stress genes essential for cell survival [60]. It is tempting to speculate that such a mechanism could contribute to the translation regulation of the stress-related selenoprotein mRNAs. This will need to be investigated.

**Figure 6:**
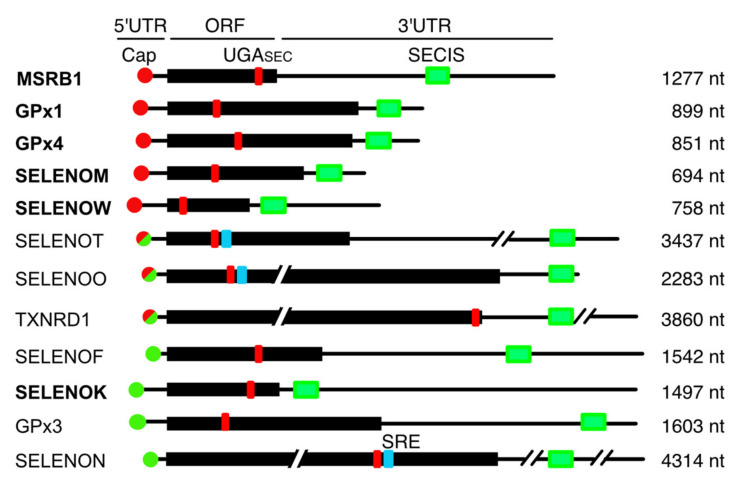
Characteristics of selenoprotein mRNAs bound by eIF3. The schematic representation of the selenoprotein mRNAs summarizes the analysis presented in Table S3. The names of the mRNAs bound by eIF3 are in bold. The open reading frames are represented by black rectangles, 5′UTR and 3′UTR by black lines. The position of the UGA_Sec_ is indicated in red. The SECIS element is in green. The Sec redefinition element (SRE) is in blue. The cap is represented by a dot. Selenoprotein mRNAs are classified according to the nature of their 5′ cap structures, as previously described [23]. Red dots: selenoprotein mRNAs with hypermethylated m_3_^2,2,7^G caps that are not recognized by eIF4E, Red/green dots: selenoprotein mRNAs with m_3_^2,2,7^G capped mRNAs that are weakly recognized by eIF4E. Green dots: m^7^G capped selenoprotein mRNAs that interact with eIF4E.

The binding of eIF3 to its target mRNAs requires the presence of stem-loop structures that have been primarily mapped in the 5′UTR but can also be found in the coding sequence or the 3′UTR. Since no selenoprotein mRNA binding sites were identified in previous PAR-CLIP experiments [29], we inspected the main features of the selenoprotein mRNAs transcripts bound by eIF3 and compared them to unbound selenoprotein mRNAs (Table S3 and Figure 6).

We examined the length of the 5′UTR, CDS, and 3′UTR, the relative positions of the UGASec codon and SECIS element, as well as the presence of conserved sequences or previously predicted structural elements [4]. No obvious sequence or structure conservation could be found in the 5′UTR of the mRNAs. In yeast, disruption of the eIF3 complex affects the translation of mRNAs with long 5′-UTR regions and whose translation is more dependent on eIF4A, eIF4B, and Ded1 but less dependent on eIF4G, eIF4E, and PABP [61]. Unlike what was observed in yeast, the size of the 5′UTR of mammalian selenoprotein mRNAs bound to eIF3 was similar to that of unbound mRNAs. A subset of selenoprotein mRNAs contain a stem-loop structure called Sec redefinition element (SRE) located adjacent to the UGA codon important for recoding in the CDS [62]. The presence of these elements, found in SELENON and predicted in SELENOT and SELENOO, does not seem to correlate with eIF3 binding. Several differences between the group of eIF3 bound and unbound mRNAs can nevertheless be pointed out (Figure 6 and Table S3). It appears that the selenoprotein mRNAs bound by eIF3 are on average three times shorter than the unbound selenoprotein mRNA (with a *p*-value of 0.011). The SECIS element is also located at a shorter distance from the ORF for eIF3-bound mRNAs, this distance is seven times greater (*p*-value of 0.11) for unbound selenoprotein mRNAs (Figure 6 and Table S3). In the 3′ UTR, the SECIS element remains the only major structural signature present in all the selenoprotein mRNAs. This raises the possibility that the SECIS element could constitute a binding platform for the recruitment of eIF3 to selenoprotein mRNAs. Alternatively, eIF3 could bind the m_3_^2,2,7^G capped of selenoprotein mRNAs via an eIF3d non-canonical driven mechanism.

Our study identified direct interactions between eIF3 and selenoprotein mRNAs. Two groups of selenoprotein mRNAs clearly emerge. A group of hypermethylated m_3_^2,2,7^G-capped mRNAs preferentially bound by eIF3 and a second group of m^7^G capped-mRNAs bound by eIF4E. Interactions of eIF3 with selenoprotein mRNAs may constitute a way to redirect mRNAs into specific translation pathways. Future studies will be required to determine if eIF3 regulates the translation of selenoprotein mRNAs and how it contributes to the mechanism of hierarchy of selenoprotein expression.

## Data Availability

Not applicable.

## Supplementary Materials

The following supporting information can be downloaded at: https://www.mdpi.com/article/10.3390/biom12091268/s1, Table S1: List of antibodies for Western blot and immunoprecipitation; Table S2: Oligonucleotides used for qRT-PCR experiments. Table S3: Analysis of selenoprotein mRNAs bound by eIF3.
