# Supplementary material for: eIF3 Interacts with Selenoprotein mRNAs
Source: Biomolecules. 2022 Sep 9;12(9):1268. doi: 10.3390/biom12091268 (PMC9496622; doi:10.3390/biom12091268)
Supplement: Supplementary file 1 [file biomolecules-12-01268-s001.zip › biomolecules-1872459-supplementary.pdf]

Table S1: List of antibodies for western blot and immunoprecipitation

Antibody	Origin	Reference
Anti-eIF3a	Polyclonal /Rabbit	Novus Biological / NBP1-18891
Anti-eIF3b	Polyclonal / Rabbit	Bethyl / A301-761A
Anti-eIF3c	Polyclonal / Rabbit	Bethyl / A300-377A-T
Anti-eIF3d	Polyclonal / Rabbit	Bethyl / A301-758A
Anti-eIF3e	Polyclonal / Rabbit	Bethyl / A302-985A
Anti-eIF3g	Polyclonal / Rabbit	Bethyl / A301-757A
Anti-eIF3i	Monoclonal / Mouse	BioLegend / 646702
Anti-eIF3k	Polyclonal / Rabbit	Novus Biological / NB100-93304
Anti-HSP90	Polyclonal / Rabbit	Santa Cruz / sc-13119
Anti-GST- HRP	Polyclonal / Rabbit	GE Healthcare / RPN1236V
HRP anti-Rabbit IgG	Polyclonal / Goat	Bethyl / A120-101P
HRP anti- Mouse IgG	Polyclonal / Goat	Bio-Rad / 170-6516

Table S2: Oligonucleotides used for qRT-PCR experiments

Target	qRT-PCR oligonucleotides
MSRB1	Fw: CAGGTTTTCCAGAATCACTTTG<br>Rev: GGCCATGGAGACGAGTGT
GPx1	Fw: TGCAACCAGTTTGGGCATCA<br>Rev: ACCGTTCACCTCGCACTTC
GPx4	Fw: CGGGCTACAACGTCAAATTCG<br>Rev: GGGGCAGGTCCTTCTCTATCA
SELENOM	Fw: TCCCGATGAGCCTCCTGTTG<br>Rev: ATGGAATGTCCTGCGTGACG
SELENOW	Fw: ACGTGGACACAGAAAGCAAG<br>Rev: ACAGCAGCCACGAGAACATC
SELENOT	Fw: TCCAGATTTGTGTTTCCTGAGG<br>Rev: CTGGGTACCGCTGGCTAATA
TXNRD1	Fw: CCTTATCATCATTGGAGGTGG<br>Rev: AAGAGGGGTGGGAGTGACAAA
SELENOF	Fw: CGGACAGTTCAACCTGCTTC<br>Rev: AATACAGGGTCTGAACCACG
SELENON	Fw: AGCTTCATCAGCACCTGGTC<br>Rev: CGGAGGTGATGTCCAAGAAG
GPx3	Fw: GGAGTACATCCCCTTCAAGC<br>Rev: CGAATGGTGCAAGCTCTTCC
SELENOK	Fw: ATCTGATTCCAGATATGATGAT<br>Rev: TGATTGATTCTACCCATTCTTC
SELENOO	Fw: GAGGAGTTTGACGCCGAGTTC<br>Rev: GCTCAGCAAGTAGAAGGTGTTTGTG
H4	Fw: AGGTGCTGCGGGACAATATC<br>Rev: GCCGAAACCATAAAGGGTGC
C-JUN	Fw: TGACTGCAAAGATGGAAACG<br>Rev: CAGGGTCATGCTCTGTTTCA
LDHA	Fw: TGGCAGCCTTTTCCTTAGAACA<br>Rev: ACGGCTTTCTCCCTCTTGCTGA
GAPDH	Fw: CTTTGGTATCGTGGAAGGACT<br>Rev: CCAGTGAGCTTCCCTTTTCAG
HPRT	Fw: TGACACTGGCAAAACAATGCA<br>Rev: GGTCCTTTTCACCAGCAAGCT
snRNA U2	Fw: TTCTCGGCCTTTTGGCTAAG<br>Rev: CTCCCTGCTCCAAAAATCCA

Table S3: Analysis of selenoprotein mRNAs bound by eIF3.

mRNA name	Ref seq	mRNA length (nts)	5'UTR (nts)	ORF position	3'UTR (nts)	UGASec position	Position of SECIS	Distance UGA-SECIS (nts)	Distance end ORF-SECIS (nts)	Type of SECIS
**MSRB1**	NM_016332.4	1277	43	44-394	883	326	871-942	545	477	2
**GPX1**	NM_000581.4	899	75	76-687	212	220	740-808	520	53	1
**GPX4**	NM_002085.5	851	50	51-644	207	267	688-764	421	44	2
**SELENOM**	NM_080430.4	694	63	64-501	193	205	537-617	332	36	2
**SELENOW**	NM_003009.4	758	83	84-347	411	120	380-468	260	33	2
SELENOT	NM_016275.5	3437	42	43-630	2807	187	3417-3422	3230	2787	2
SELENOO	NM_031454.2	2283	26	27-2036	247	202	2150-2228	1948	114	2
TXNRD1	NM_001093771.3	3860	24	25-1974	1886	1966	2189-2275	223	215	2
SELENOF	NM_004261.5	1542	15	16-513	1029	301	1103-1184	802	590	2
**SELENOK**	NM_021237.5	1497	71	72-356	1141	345	475-562	130	119	2
GPX3	NM_002084.5	1603	64	65-745	858	281	1395-1471	1114	650	2
SELENON	NM_020451.3	4314	38	39-1811	2503	1422	2905-2972	1483	1094	1

Average results	mRNA length	5'UTR	ORF lenght	3'UTR	UGASec position	Position of SECIS	Distance UGA-SECIS	Distance end ORF-SECIS
**eIF3 bound mRNAs**	996	64	423	489	247	615	366	127
unbound mRNAs	2839	38	1249	1574	726	2193	908	908
p-value (bound/unbound)	0.011	0.011	0.039	0.052	0.19	0.42	0.049	0.11
**Ratio unbound/bound**	2.8	0.6	2.9	3.2	2.9	3.6	2.5	7
